# Ecotoxicity of Copper(I) Chloride in Grooved Carpet Shell (*Ruditapes decussatus*)

**DOI:** 10.3390/antiox11112148

**Published:** 2022-10-30

**Authors:** Giuseppe Esposito, Paolo Pastorino, Marino Prearo, Gabriele Magara, Alberto Cesarani, Rosa Freitas, Barbara Caldaroni, Domenico Meloni, Antonio Pais, Alessandro Dondo, Elisabetta Antuofermo, Antonia Concetta Elia

**Affiliations:** 1 The Veterinary Medical Research Institute for Piemonte, Liguria and Valle d’Aosta, 10154 Torino, Italy; 2 Department of Chemistry, Biology and Biotechnology, University of Perugia, 06123 Perugia, Italy; 3 Department of Agriculture, University of Sassari, 07100 Sassari, Italy; 4 Department of Animal and Dairy Science, University of Georgia, Athens, GA 30602, USA; 5 Department of Biology and CESAM, University of Aveiro, 3810-193 Aveiro, Portugal; 6 Department of Veterinary Medicine, University of Sassari, 07100 Sassari, Italy

**Keywords:** antioxidant biomarkers, bivalve, clam, invertebrates, mollusks, trace elements

## Abstract

Copper (Cu) is a ubiquitous trace element in the aquatic environment, and is usually found at low levels. Copper environmental concentrations can be altered as a result anthropogenic activities. Shellfish are useful bioindicators to ensure adequate environmental monitoring. Thus, the aim of the present study was as follows: (a) determine the LC_50_ of copper(I) chloride in grooved carpet shell (*Ruditapes decussatus*) collected in the Santa Gilla lagoon (Sardinia, Italy), and (b) analyze the antioxidant biomarkers in digestive gland and gills of same specimens exposed to different concentrations of the above-mentioned metal (0.045, 0.45, and 0.90 mg/L) for 96 h. A withdrawal period of 96 h was considered for the treated clam, carrying out the same biochemical analyses, superoxide dismutase (SOD), catalase (CAT), selenium-dependent glutathione peroxidase (Se-GPx), glutathione S-transferases (GSTs), and total glutathione (GSH+2GSSG) in the two tissues. Different time and dose responses of the antioxidant biomarkers were recorded in the digestive glands and gills. Oxidative stress biomarkers highlighted the ability of Cu to induce oxidative stress in *R. decussatus*. Clam, following the withdrawal period of 96 h, has not been able to achieve the control levels of all biochemical markers in the digestive gland and gills. *R. decussatus* can be a suitable model to assess the ecotoxicity of copper in aquatic ecosystems. These findings may advance knowledge on the role and the effects of copper on oxidative stress biomarkers in grooved carpet shell. The metal ecotoxicity response can be useful to perform accurate biomarker-based monitoring programs using this bivalve species.

## 1. Introduction

Copper (Cu) is a reddish-brown chemical element, a ductile and malleable metal, belonging to Group 11 (IB) of the periodic table. This metal is usually found in the environment both in a wide variety of mineral salts and organic compounds and in the metallic form [1]. Copper is slightly soluble in water, salt, or mildly acidic solutions, but it can be dissolved in nitric and sulfuric acids, as well as basic solutions of ammonium hydroxide or carbonate [1].

As the use of metals has increased significantly over the years, the increased likelihood of their accumulation and its impacts at various trophic levels is expected. Copper is one of the most actively traded commodities, used worldwide mainly for electrical applications, but also in architecture, automotive, electrical, fuel gas, marine products, and telecommunications, etc. [2]. Thus, it has several fields of application, namely in agriculture, as Cu-compounds (e.g., copper chloride (CuCl_2_), copper hydroxide (Cu(OH)_2_), copper oxide (Cu_2_O), etc.) are commonly used as antimicrobials for foliar disease management [3]. In 2020, global refined copper usage stood at nearly 25 million metric tons, with an increase of 2.3% from the previous year [4]. Therefore, the release of Cu and its compounds into the environment occurs mainly through mining [5,6,7], smelting facilities [8,9,10], and urbanized areas [11,12,13]. However, accidental release into water bodies can also occur through commercial antifouling (AF) paints in order to prevent the growth of encrusting organisms [14,15,16,17].

Naturally abundant in the Earth’s crust and surface waters as a pure metal, copper has a high thermal and electrical conductivity. Copper compounds are generally found as copper (II) salts, with low levels in the aquatic environment. According to Nordberg and co-authors [18], seawater concentrations are usually less than 0.001 mg/L, but higher values were reported by Hall and Anderson [19] in different European saltwater environments. In particular, the range of reported values was highly variable, depending on the area: marinas (0.0003–0.061 mg/L), harbors (0.00014–0.0207 mg/L), coastal/open sea areas (<0.00001–0.086 mg/L), and estuaries (0.00006–0.0141 mg/L) [19]. Harbors and marinas represent a source of Cu, where AF paints contribute to the accumulation of this metal both in water and sediment. In the water column, dissolved Cu concentrations can range from 0.0027 ± 0.0037 mg/L to 0.0014 ± 0.0018 mg/L (marinas and harbors, respectively) [20]. In the sediment, these paints can give rise to small Cu-based fragments, also known as antifouling paint particles (APPs), toxic to sediment-dwelling biota as bivalves [21]. In marine and estuarine areas, published studies have demonstrated that Cu can accumulate in inhabiting wildlife species, including in bivalves: 1 mg/kg wet weight (ww) in clams *Ruditapes philippinarum* and *R. decussatus* from the Ria de Aveiro, Portugal [22]; 0.7 mg/kg ww in common edible cockle (*Cerastoderma edule*) from the same coastal lagoon [23]; an average of about 2 mg/kg ww in grooved carpet shell (*R. decussatus*) from Sardinian lagoons [24]; an average of roughly 15, 13, and, 12 mg/kg dry weight (dw) in European razor clam (*Solen marginatus*), Japanese carpet shell (*R. philippinarum*), and olive green cockle (*C. glaucum*), respectively [25] from Venice lagoon, and an average of about 112 mg/kg ww in Pacific cupped oyster (*Crassostrea gigas*) from different areas of the North Adriatic [26].

The increasing use and release of Cu into aquatic environments have been identified as the cause of adverse effects on aquatic organisms at cellular and individual levels [27], including in marine sessile invertebrates [28]. Among them, bivalves have evolved subcellular strategies for the accumulation, regulation, and immobilization of excess essential and nonessential trace elements [29]. Exposure to environmental pollutants results in uptake through their ability to filter water (filter-feeders), and significant amounts of chemical compounds can be accumulated within their organs, without any apparent adverse effect [30]. In fish, increased Cu exposure can activate the detoxifying response, particularly through increased glutathione and metallothionein concentrations, as well as through an impaired antioxidant enzymatic activity and cellular damage [31]. Regarding shellfish, a study conducted on shark fin mussel (*Hyriopsis bialatus*) showed that Cu exposure caused imbalances in the main oxidative stress biomarkers, such as superoxide dismutase, catalase, glutathione peroxidase, and glutathione S-transferases [17].

The published literature has already demonstrated the toxicity of metals, including Cu, caused in different bivalve species, with harmful effects in Pacific cupped oyster (*C. gigas*) exposed to 50 μg/L of copper (II) chloride (CuCl_2_) [32]. Copper can also lower the concentration of glutathione in Mediterranean mussel (*Mytilus galloprovincialis*) [33], whereas the activity of glutathione S-transferases in the same species and in Asian green mussel (*Perna viridis*) was increased [34,35].

The clam species *R. decussatus* (Linnaeus, 1758)*,* known as the grooved carpet shell, is among the most suitable organisms to be used as a biological indicator in order to assess the changes in environmental quality [24]. In addition, extensive biological knowledge exists about this species, which is suitable for the study of physiological, immunological, and behavioral alterations that environmental pollutants might cause [36].

*R. decussatus* is endemic to the Mediterranean Sea, and is also distributed along the Atlantic coasts (from Norway to Senegal) and along the southern and western coasts of the British Isles [37,38]. This species is generally found in sheltered bays, estuaries, ponds, and lagoons (i.e., transitional waters) where it lives burrowed in sand, muddy gravel, clay, or silty mud (0–1 m).

Shellfish farming is currently the main production item in Italian aquaculture, and Sardinia contributes to the national sector with respectable average annual quantities. In 2008, the production of mussels and oysters alone was close to 11,000 tons, with a commercial value estimated at around 20 million Euros [39]. However, these data do not give an idea of the considerable overall production of molluscs (mainly bivalves) that characterises Sardinia. They do not include the production linked to the simple harvesting in coastal brackish environments of other species that are particularly appreciated and abundant, such as warty venus (*Venus verrucosa*), smooth callista (*Callista chione*), olive green cockle (*C. glaucum*), and, above all, clams (*R. decussatus*). The latter is especially the flagship Sardinian production due to consumers’ excessive care, despite a rather high market price (≈20–25 €/kg) to their congener (*R. philippinarum*), for which the cost is lower (≈10–18 €/kg) and whose main Italian production areas are located in the North Adriatic [40].

The aim of the present study was to investigate the effects of copper(I) chloride on the health status of *R. decussatus* by investigating the biochemical changes of the antioxidant and detoxifying biomarkers in the target organs (i.e., gills and the digestive gland), during both the copper treatment and copper withdrawal.

## 2. Materials and Methods

### 2.1. Study Area

**Figure 1:**
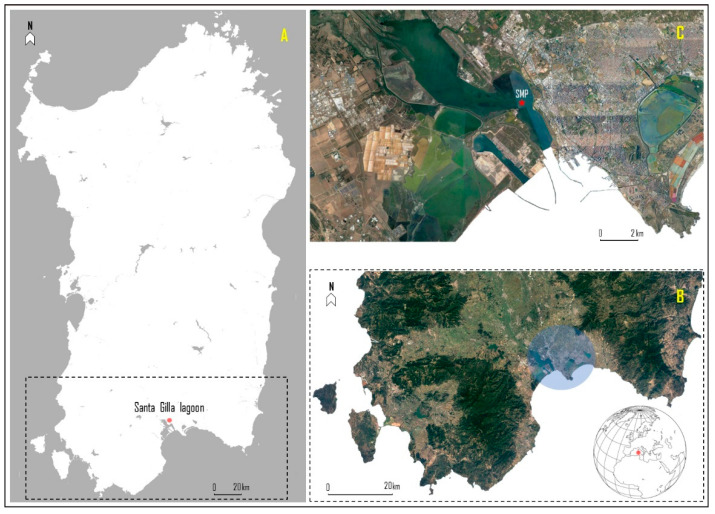
Study area: (**A**) = Sardinia Island, south of the sampling area; (**B**) = in the blue circle is identified the Santa Gilla lagoon; (**C**) = details of the lagoon, and sampling molluscs point (SMP; red star).

The Santa Gilla lagoon is one of the two largest wetlands located near the city of Cagliari (South Sardinia, Italy; 39°14′36.69″ N, 9°02′24.73″ E), with a surface area of about 1800 hectares (Figure 1). Located in the southern part of the Campidano plain, it is fed by the Riu Cixerri and the Flùmini Mannu, as well as other lesser waterways and drainage canals. However, the water exchange, provided by the rivers, is faster in the southern basin than in the innermost areas [41]. However, over the years, lagoon improvements and modifications have resulted in a reduction in these inflows, with direct effects on benthic biocoenosis, as well as salinity [42,43]. The lagoon—with a mainly sandy-muddy bottom—is about 400 m wide and the exchange with the sea waters takes place through the “Sa Scafa” canal, which has a maximum depth of about −2 m. Generally, the lagoon has fairly variable physicochemical parameters depending on the area, i.e., salinity of 31.23 ± 4.34, pH of 7.91 ± 0.24, and dissolved oxygen concentration of 8.71 ± 3.16 mg/L [43]. 

Overall, it is a large area strongly influenced by an anthropogenic load that regulates water turnover and limits area development. The lagoon mainly received industrial and municipal sewage discharge, although also influenced by other sources of anthropogenic pollution (e.g., airports and railways). According to previous reports, this wetland is considered a moderately polluted environment [44] with low concentrations of copper in sediment (29 ± 16 mg/kg [43]; 29 ± 7.6 mg/kg [45]), in water (0.002 ± 0.0013 mg/L [45]) and in shellfish (highest value among lagoons of 4.8 ± 1.7 mg/kg ww [24]), which are an important local economic resource.

### 2.2. Sampling and Acclimation Phase

In 2018, wild specimens of grooved carpet shell (*Ruditapes decussatus*) were manually sampled in a natural bed at roughly 1 m depth. The sampling point (SMP; 39°22′87.97″ N, 9°07′80.72″ E) was well-defined within the classified harvesting area [46] (Figure 1C). A total of 10 kg ww of clams were sampled (approx. 1000 bivalves). Then, after careful inspection (e.g., morphometric analysis and the absence of shell lesions), the suitable specimens were stored, dry, in a cool box (mean length and mean wet weight of 25 ± 0.8 mm and 9.0 ± 1.4 g, respectively), and were transported to the laboratory for depuration and acclimation. For 15 days, the clams were divided into two 100 L tanks (35 × 85 × 45 cm) with a RAS system (Recirculating Aquaculture System) and artificial seawater (reverse osmosis water plus artificial salt-Salinity^™^ by Seachem Aquavitro^®^). The clams were maintained with a natural photoperiod (i.e., light through windows) at constant parameters to mimic the sampling conditions: salinity (33), dissolved oxygen (9.8 ± 1.2 mg/L), temperature (26 ± 0.1 °C), pH (8.0 ± 0.08), and hardness (8.2 ± 0.5 dKH). 

Water was renewed daily, and during the second week, the clams were fed daily with 500 mL per tank of *Isochrysis galbana* and *Dunaliella tertiolecta*, at an approximate concentration of 5 and 12 × 10^6^ cells/mL, respectively.

### 2.3. Experimental Design

#### 2.3.1. Preparation and Quantification of Copper in Water

The involved copper(I) chloride (CuCl) (Sigma-Aldrich^®^; SKU code: 229628) was provided as a dry powder, at least 99% pure. Copper stock suspensions were prepared at nominal metal concentrations of 1.875, 3.75, 7.5, 15, and 30 mg/L (for the determination of lethal concentration; Section 2.3.2), and 0.045, 0.45, and 0.9 mg/L (sub-acute trial; Section 2.3.3) in deionized water and ultrasonicated to split possible clusters of particles (40 W, 4 min; S-450 Ultrasonifier, Branson Ultrasonics Corporation, Danbury, CT). The chemical stock solutions were stored at a controlled temperature (≥20 °C) in the dark.

To confirm the dosing concentrations in water, samples from each tank and from both assays were collected daily after the re-establishment of Cu concentrations and were pooled (45 mL/tank, i.e., *n* = 3 replicates/treatment of 15 mL each) plus the control (0 mg/L) (Supplementary Table S1). The concentrations of Cu were quantified by inductively coupled plasma mass spectrometry (ICP-MS Xseries II, Thermo Scientific, Bremen, Germany) according to a method already described by Søndergaard et al. [47]. The limit of quantitation of the method (LOQ) was 0.010 ppm (0.010 mg/L).

#### 2.3.2. Lethal Concentration (LC_50_)

The pre-experimental phase consisted of 18 independent plastic tanks (30 × 23.5 × 34.5 cm), with three tanks/replicates per treatment, each containing 20 L of seawater reconstituted at a salinity of 33. In each tank, both oxygenation and recirculation were provided by an air stone and an immersion pump, respectively (flow rate = 200 L/h; power = 1 W, size = 4.4 × 4.1 × 3.7 cm). The pre-experimental phase was carried out in triplicate and for 96 h of exposure. The main physicochemical parameters were kept constant throughout the whole period (see Section 2.2). Each tank housed 20 specimens of grooved carpet shell (*Ruditapes decussatus*) exposed to different increasing CuCl concentrations: 1.875, 3.75, 7.5, 15, and 30 mg/L plus control (0 mg/L). The test exposure concentrations were selected considering pilot tests to define the correct dilution range, as well as the available references [48]. Concentrations that induced the LC_50_ of the exposed samples were calculated by statistical interpolation from experimental data using the US EPA Toxicity Relationship Analysis Program (TRAP version 1.30), with a Gaussian distribution and logarithmic transformation of exposure variables sized for ecotoxicological tests.

Overall, the following operations were carried out daily: monitoring of water parameters, control of survival, total water renewal, and re-establishment of CuCl concentrations. The clams were fed daily with approximately 100 mL per tank of *Isochrysis galbana* (Parke, 1949) and *Dunaliella tertiolecta* (Butcher, 1959) (5 and 12 × 10^6^ cells/mL, respectively).

#### 2.3.3. Sub-Acute Trial and Sampling Procedures

The experimental trial consisted of 12 independent plastic tanks, three per treatment, (30 × 23.5 × 34.5 cm), each containing 20 L and 40 clams. The characteristics of the aquaria and the physicochemical parameters, as well as the procedures, were the same as those highlighted in Section 2.2 and Section 2.3.2. The experimental trial was carried out in triplicate for eight days, divided into two phases: four days of exposure (96 h) and four days of metal withdrawal (96 h) without contaminant. During the first phase (exposure), *Ruditapes decussatus* specimens were exposed to CuCl concentrations of 0.045, 0.45, and 0.90 mg/L (selected according to the LC_50_ pre-assay; see Section 2.3.2) plus the control (0 mg/L).

From each tank, three specimens were sampled daily at the same time in the two phases (*n* = 9 specimens per treatment). Immediately, bivalves were dissected with a scalpel after cutting the adductor muscles and, after being separated from the shell, the gill tissues and digestive glands were taken. Subsequently, the tissues from each tank were pooled (*n* = 3 samples/tissue/treatment) and stored at −80 °C for he biochemical analyses.

### 2.4. Oxidative Stress Biomarkers in Clams

The biomarker absorbance was measured in triplicate by spectrophotometry (Varian Cary 50) at 25 °C. A fraction of the digestive gland (0.03 g) and gills (0.06 g) was used for the biochemical analysis. The cytosolic extraction was performed as previously reported on the bivalve [49].

For the total glutathione (GSH+2GSSG) content determination, tissues were homogenized in 5% sulfosalicylic acid, 4 mM EDTA, and centrifuged at 30,000× *g* for 3 min (4 °C), following the method of Akerboom and Sies [50]. Absorbance was recorded at 412 nm using a 100 mM potassium phosphate buffer (pH 7), 1 mM EDTA, 1 unit of GR, 4 mg/mL NADPH, and 1.5 mg/mL 5,5′-dithiobis(2-nitrobenzoic acid) (DTNB), both dissolved in 0.5% NaCO_3_, and glutathione disulphide was used as the reference. The total GSH was expressed in nmol/g tissue.

For the enzyme activities, the tissues were homogenized on ice, subject to dilution (1:5) in 100 mM pH 7.5 KP buffer, containing 2.5% NaCl, 0.1 mg/mL Bacitracin, and 0.008 TIU/mL Aprotinin. The samples were centrifuged at 18,000× *g* for 20 min at a temperature of 4 °C. Appropriate volumes of cytosolic fraction were then used for the different assays. The superoxide dismutase (SOD) activity was performed using a 50 mM Na_2_CO_3_ buffer of pH 10 with 0.1 mM EDTA, 500 mM cytochrome C, 1 mM hypoxanthine, and xanthine oxidase (550 nm) [51]. The catalase (CAT) levels were assessed using a sodium phosphate buffer of 100 mM pH 7 and 24 mM H_2_O_2_ (240 nm) [52]. The selenium-dependent glutathione peroxidase (Se-GPx) activity was measured in a sodium phosphate buffer of 100 mM pH 7.5 added with EDTA 1 mM, NADPH 0.12 mM, GSH 2 mM, glutathione reductase 1U, and 0.6 mM H_2_O_2_ (340 nm) [53]. The glutathione S-transferases (GSTs) levels were assessed using sodium phosphate buffer of 100 mM pH 6.5 using GSH and CDNB 2 mM (340 nm) [54].

The cytosolic total protein concentration was determined following the method of Lowry et al. [55] and was used to express the activity of SOD (U/mg prot), CAT (µmol/min/mg prot), Se-GPX, and GSTs (nmol/min/mg prot).

### 2.5. Statistical Analysis

Descriptive statistics of the considered biomarkers of oxidative stress were performed (Supplementary materials, Table S1). The biomarkers measured in the grooved carpet shell (*Ruditapes decussatus*) collected in the Santa Gilla lagoon (Sardinia, Italy) were compared and analyzed with a general linear model (GLM). The model was defined as follows:

(1) $$activity/concentration_{ijk}=\mu +time_{i}+dosage_{k}+time_{i}*dosage_{k}+\varepsilon_{ijk}$$

where “*activity/concentration*” was the activity of the five considered biomarkers (i.e., CAT, GSH+2GSSG, GSTs, Se-GPx, and SOD); “*time*” is the cross-classified fix effect of the *i-th* phase (*i* = 2 levels, exposure and recovery); “*dosage*” is the cross-classified fix effect of the *k-th* considered copper(I) chloride (CuCl) (*k* = 4 levels; control, 0.045, 0.45 and 0.90 mg/L); “time * dosage” is the interaction between the *i-th* phase and the *k-th* considered CuCl (32 levels); “ε” is the residual error. All of the basic statistics and analyses were carried out using R software (v. 4.0.5; RStudio Team, 2021).

## 3. Results

The obtained LC_50_ value was equal to 3.7 ± 0.2 mg/L of copper(I) chloride. However, lower concentrations of this value were selected for the sub-acute trial (see Section 2.3.3), relevant from an environmental point of view and based on preliminary data obtained in previous studies for the same study area [24]. The confirmatory dosing copper concentration data in water samples for both assays are also shown in Supplementary Table S1.

In addition, the descriptive statistics are shown in Supplementary Table S2, whereas the results of the linear model are reported in Table 1.

**Figure 2:**
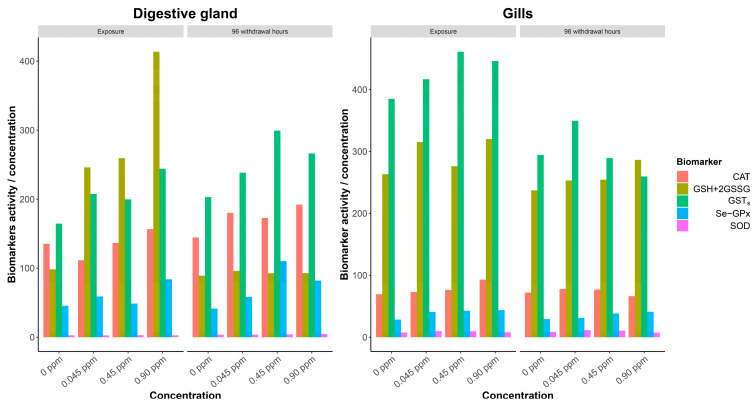
Biomarker estimated levels in the digestive gland and in the gills (**left** and **right**, respectively).

The concentration of copper(I) chloride (CuCl) showed a significant effect on the CAT, Se-GPx, and GSTs measured in the digestive gland and on the SOD and GSTs measured in the gills. The three biomarkers in the digestive gland (CAT, Se-GPx, and GSTs) showed increasing activities along the increasing exposure gradient; all of the three enzymes showed the highest activity when the CuCl concentration reached 0.90 mg/L (Figure 2). An opposite trend was observed for the gills, where the SOD and GSTs decreased their activities from the control to the higher concentration; both enzymes showed the highest activity at the 0.045 mg/L CuCl concentration (Figure 2). Finally, the interaction between time and concentration was highly significant (*p* < 0.001) only for the Se-GPx measure in the digestive gland and on the SOD measured in the gills (Table 1).

Time (i.e., the difference between exposure and recovery phase) was highly significant (*p* < 0.001) for all of the investigated enzymes measured in the digestive gland: SOD, CAT, Se-GPx, and GSTs increased their activities moving from the exposure to the recovery phase, whereas the GSH+2GSSG content decreased, i.e., 120.37 during exposure and 76.41 during recovery, respectively (Table 1 and Figure 2). As far as the biomarkers measured in the gills, time was found to be significant for SOD and Se-GPx, which increased their activities from exposure to recovery, and for GSTs, which decreased their activity (Table 1 and Figure 2).

The enzymatic activities were also analyzed daily (Supplementary Table S2). Overall, the highest activities of biomarkers in the digestive gland and in the gills were observed on day 3 and day 1, respectively. However, a clear pattern among the five investigated biomarkers was not observed. In fact, looking at each specific biomarker in the digestive glands, the highest values were observed on day 3 (GSH+2GSSG), day 6 (SOD, CAT and Se-GPx), and day 7 (GSTs). As far as the gills, the highest values were observed on day 1 (CAT, Se-GPx, and GSTs), day 2 (GSH+2GSSG), and day 8 (SOD).

## 4. Discussion

Copper is an essential element for aquatic and human life [56], although at high concentrations it can be toxic. Biochemical, photochemical, or thermochemical processes can lead to the formation of a Cu(I) dissolved concentration in seawater [57]. At constant salinity and pH, the inorganic complexation of Cu remains constant, although variations in metal speciation may be related to the presence of organic ligands such as dissolved organic matter (DOM) [58,59,60,61,62]. However, the sources, distribution, and nature of organic ligands remain unclear [63].

In the present study, cuprous ion (Cu^+^) induced changes in the antioxidant and detoxifying pathway of *Ruditapes decussatus* exposed to sublethal concentrations of copper(I) chloride. The analyses of the levels of the main oxidative stress biomarkers were carried out in the gills and the digestive gland, as they have a high potential for reactive oxygen species (ROS) production [64,65]. Gills play a respiratory role in mollusks, and they represent the tissue most in contact with the external environment, whereas the digestive gland represents the main organ involved in the detoxification of toxic substances, including metals [66].

In this study, biomarker responses were related to copper concentrations, especially noticed in the digestive gland, highlighting the capacity of Cu to induce oxidative stress in *R. decussatus*. The results obtained further demonstrated the different biochemical pattern between organs, with a higher antioxidant capacity in the digestive gland, especially after the exposure period, while the increased detoxification capacity was more pronounced in the gills.

Glutathione is one of the most powerful biological antioxidant molecules in mussels [49] and the reduced form of the thiol (GSH) is well recognized as one of the most potent intracellular copper ligands in aquatic vertebrates and invertebrates [17,31]. In this study, the digestive gland was the most involved tissue in the modulation of the total glutathione levels. Recent data showed that GSH levels increased significantly in the digestive glands of freshwater mussel (*Unio mancus*) exposed to antifouling copper pyrithione, while thiol levels were unchanged in the gills [67]. These findings are in agreement with previous research in which the key role of the digestive gland in metal bioaccumulation and detoxification was discussed [68]. A late increase in total glutathione levels was measured in the digestive gland of *R. decussatus*. Similar results were observed in the liver of zebrafish (*Brachydanio rerio*) exposed to sublethal concentrations of copper sulfate; the authors supposed an adaptive response of the experimental model to copper treatment suggested by the increased levels of glutathione content, catalase, and glutathione S-transferases [69]. However, the unchanged thiol concentration recorded in the digestive gland during the exposure phase (day 1 and 2) may be related to the complex defense mechanism carried out by glutathione against Cu, as previously shown in fish and bivalve [17,31]. This mechanism involves a series of intermediate redox reactions ending in the transfer of the metal to metallothionein (MT), a further protective system against metal toxicity that activates more slowly than glutathione [17,31]. In detail, when in presence of a cuprous ion, GSH can be quickly overproduced and promptly bind to the metal forming a GSH-Cu^+^ complex. This complex can undergo oxidation in the presence of oxygen, forming the superoxide radical (O_2_^−^) and the intermediate GSSG-Cu^+^ complex, which is further oxidized to Cu^2+^-GSSG. Then, this complex binds to metallothionein, forming a GS-Cu-MT intermediate and, finally, glutathione can be released [70]. As previously reported in fish and bivalves exposed to copper(I) chloride [17,31], the analytical method used to measure the total glutathione is not able to detect the glutathione-copper complexes, resulting in unchanged levels of total glutathione. This hypothesis is supported by the late increase in glutathione levels (three days) of Cu exposure, when copper was likely transferred to MT, as reported in previous studies on fish and bivalves [17,31]. During the detoxification phase, the levels of total glutathione in the gills and digestive glands were compared with the control, suggesting a slackening of the stress-inducing pressure driven by copper.

The GSTs activity increased in the digestive gland of the specimens exposed to the two higher Cu concentrations, while the opposite trend was observed in the gills. These findings are in agreement with previous published data on Asian clam (*Corbicula fluminea*) exposed to 10 μg Cu/L, in which the GSTs responses fluctuated between a 10-fold decrease to a 4-fold increase in the gills and digestive gland, respectively [71]. The different enzyme activity measured in the present study between the gills and digestive glands may be related to the different exposure and physiological functions of the two tissues. Gills comprise an external facing organ directly in contact with the environmental medium and play a key role in the bioconcentration process of contaminants into aquatic organisms. They play a respiratory and not mainly detoxifying role, thus the reduction in GSTs activity following exposure to high concentrations of copper is not surprising. On the contrary, the digestive gland is the tissue responsible for detoxification and the enhancement of the detoxifying activity in the presence of copper was expected, suggesting a boost of the detoxifying shield of the bivalve against copper.

The CAT and Se-GPx activity was higher in the digestive gland, but no differences were noticed between the exposure and recovery periods. Comparing the levels of GSTs, CAT, and Se-GPx, the data suggest that *R. decussatus* can change the GSTs activity faster from exposure to a recovery in comparison with CAT and Se-GPx. This can be related to the time that these enzymes need to change performance, advising for a long time to change the activity of these enzymes on the bivalve. CAT and Se-GPx can transform the harmful hydrogen peroxide (H_2_O_2_) in H_2_O, and they are strictly related to the activity of SOD, which can dismutate O_2_^−^ in H_2_O_2_. Previous studies showed increased activities of CAT and Se-GPx in the gills of Mediterranean mussel (*Mytilus galloprovincialis*) exposed to 10 μg CuO nanoparticles/L [72]. Similar results were also observed in common carp (*Cyprinus carpio*) exposed to nanoparticles of the same compound [73]. In the present study, the unchanged SOD levels in the gills of copper-treated mussels compared with the control group concomitant with the increased levels of Se-GPx in both tissues and CAT in the digestive gland during the intoxication phase advise for an enzyme-independent overproduction of hydrogen peroxide. This outcome can be explained by the ability of the GSSG–Cu^+^ complex to convert the superoxide ion into hydrogen peroxide in a SOD-like manner when oxidizing the Cu^2+^-GSSG complex [74]. At day 2, we observed low Se-GPx levels compared with the untreated mussels, following an unchanged CAT activity. This may suggest a compensation mechanism between the two antioxidant enzymes. This is not an unusual mechanism as it has already been reported in aquatic organisms exposed to metals [75,76]. However, lower CAT levels observed in clam gills exposed to a higher concentration of copper(I) chloride at day 3, concomitant with a decreased GSTs activity, indicates a weakening of the antioxidant shield against copper toxicity. The effects of copper(I) chloride treatment on CAT were already reported in freshwater fish and threatened mussels, associating the enzyme inhibition with an interplay between the iron and copper in CAT structure [31]. In detail, iron can be displaced by copper from the iron–sulfur clusters of CAT when the copper concentration is higher than the physiological levels [77].

During the detoxification phase, the enhancement of both the antioxidant and detoxifying pathways, and especially of Se–GPx, in the digestive gland of specimens exposed to copper suggests a boost in the antioxidant defense of *R. decussatus* and an attempt of this clam species to counteract the stress pressure induced by the higher concentrations of cuprous ion. The decrease of SOD activity in the gills of clams exposed to a higher CuCl concentration until day 7 may be explained by a mechanism of interplay between copper and zinc. Decreased levels of SOD activity were previously reported by Elia et al. [17] in shark fin mussel (*Hyriopsis bialatus*) exposed to sublethal concentrations of copper(I) chloride. Similarly, studies have shown a decreased level of SOD activity in the digestive gland and gills of *M. galloprovincialis* exposed to copper 0.079–25 μM for seven days [78] and in the gills of deep-sea hydrothermal vent mussel (*Bathymodiolus azoricus*) exposed to 0.4 μM Cu for 24 h [79]. Indeed, one cupric and one zinc ion are present in each sub-unit of cytosolic SOD [80]. We may assume that, once exposure to Cu was stopped, glutathione and the other trapping metal macromolecules released the chelated cupric and cuprous ions. As a consequence, the concentration of free copper in the tissue increased and the ratio of copper/zinc changed, favoring the displacement of zinc by copper in SOD subunits. Furthermore, because of the hepatic increased activity of Se–GPx and GSTs levels, an overproduction of ROS cannot be excluded.

## 5. Conclusions

A dose–time dependent antioxidant response to Cu exposure was observed in the digestive gland and gills of *Ruditapes decussatus*, suggesting that metal can induce oxidative stress in both tissues. This study brings new knowledge about the stressful effects of Cu on aquatic organisms. Therefore, oxidative stress biomarkers can be important tools in biomonitoring programs as they allow for detecting the effects of copper on this mollusk species in its environment. However, because Cu dynamics are rather complex, further studies are needed to fully understand the pro-oxidant role of monovalent copper in aquatic invertebrates.

## Figures and Tables

**Table 1:** Estimated means of the biomarkers used during copper treatment and post copper treatment and in the four different concentrations.

Organ	Phase		Copper Treatment (CuCl mg/L)				SEM	Phase·mg/L
Biomarker	Exposure	96 Withdrawal Hours	Control	0.045	0.45	0.90		
**Digestive Gland**								
CAT	94.76 ^B^	**133.47** ^A^	109.43 ^ab^	104.46 ^b^	118.70 ^ab^	**123.88** ^a^	3.09	NS
GSH+2GSSG	**120.37** ^A^	76.41 ^B^	75.10	101.96	105.43	11.05	5.88	NS
GSTs	139.95 ^B^	**178.74** ^A^	134.52 ^b^	150.96 ^ab^	173.28 ^a^	178.61 ^a^	5.07	NS
Se-GPx	36.03 ^B^	**50.13** ^A^	30.58 ^B^	36.59 ^B^	51.50 ^A^	53.64 ^A^	2.01	***
SOD	2.18 ^B^	**2.93** ^A^	2.33	2.49	2.66	2.74	0.07	NS
**Gills**								
CAT	**55.56**	51.82	55.45	54.99	54.18	50.12	1.51	NS
GSH+2GSSG	**214.39**	213.10	202.75	207.85	219.25	225.12	3.85	NS
GSTs	**318.72** ^A^	238.48 ^B^	289.58 ^a^	309.54 ^a^	281.23 ^ab^	234.03 ^b^	8.35	NS
Se-GPx	20.21 ^b^	**25.36** ^a^	21.58	21.25	23.71	24.61	0.88	NS
SOD	5.27 ^B^	**6.56** ^A^	6.14 ^A^	6.84 ^A^	6.09 ^A^	4.58 ^B^	0.22	***

Different capital (*p* < 0.001) or small (*p* < 0.01) letters within a row (biomarker) indicate significant differences among phases or concentrations. No letters mean no significant differences among the treatments. *** = *p* < 0.001, and NS = not significant. The highest value for each biomarker is identified.

## Data Availability

Data are contained with the article and supplementary materials.

## Supplementary Materials

The following supporting information can be downloaded at: https://www.mdpi.com/article/10.3390/antiox11112148/s1, Table S1: Determination of dosing Cu-concentrations in water samples of both assays; Table S2: Descriptive statistic of biomarkers.
